# Assessing the Prognostic Value of the Neutrophil-to-Lymphocyte Ratio in Stage I Non-Small-Cell Lung Cancer with Complete Resection

**DOI:** 10.1155/2022/6837872

**Published:** 2022-06-22

**Authors:** Wei Liu, Tiantian Zhang, Li Li, Jue Zou, Chunhua Xu

**Affiliations:** ^1^Department of Respiratory Medicine, Affiliated Nanjing Brain Hospital, Nanjing Medical University, Nanjing, Jiangsu 210029, China; ^2^Clinical Center of Nanjing Respiratory Diseases and Imaging, Nanjing, Jiangsu 210029, China; ^3^Affiliated Nanjing Brain Hospital, Nanjing Medical University, Nanjing, Jiangsu 210029, China

## Abstract

**Purpose:**

To explore the prognostic value of the neutrophil-to-lymphocyte ratio (NLR) in stage I non-small-cell lung cancer (NSCLC) undergoing surgery. *Patients and Methods*. Between 2014 and 2016, a total of 190 patients with postoperative pathology of stage I NSCLC who underwent radical surgery at Nanjing Chest Hospital were studied. Clinical data were analyzed and classified into low-risk, moderate-risk, and high-risk groups based on independent risk factors to assess the prognosis.

**Results:**

NLR was associated with histological type and gender, and patients with an elevated NLR have poor overall survival (OS). Lymphovascular invasion, red blood cell distribution width-standard deviation (RDW-SD), and carcinoembryonic antigen (CEA) were independent prognostic factors for progression-free survival (PFS) in postoperative patients with stage I NSCLC, while NLR, RDW-SD, and CEA were independent risk factors for OS. Both PFS and OS were shorter in the low-risk group than in the medium-risk and high-risk groups.

**Conclusions:**

NLR, RDW-SD, CEA, and lymphovascular invasion are independent risk factors for postoperative prognosis in patients with stage I NSCLC, and the combination has a predictive value.

## 1. Introduction

Lung cancer is one of the most common tumors in the world, accounting for 12% in men and 13% in women, and approximately 80% of lung cancers are NSCLC, with an overall 5-year survival rate of only 21% [1]. With the use of low-dose multilayer spiral CT, the screening rate of patients with early lung cancer has been improved and the mortality rate has been reduced by 20%, but there is still a risk of recurrence and even death in early-stage lung cancer patients following surgery [2]. Adopting a convenient and economical auxiliary diagnostic method can help us find the patients with early lung cancer who are prone to recurrence and death, so as to improve the survival and prognosis by early intervention. In NCCN 2021, it is stated that postoperative adjuvant therapy is not recommended for stage IA NSCLC, and postoperative adjuvant chemotherapy is recommended for patients with stage IB NSCLC with high-risk factors (high-risk factors include neuroendocrine tumors, lymphovascular or visceral pleural invasion, wedge resection, tumor diameter ≥4 cm, and unknown lymph node status) [3]. In the real world, postoperative recurrence and death are equally present in stage IA patients without the aforementioned high-risk factors. Several studies have shown that inflammatory biomarkers such as NLR, lactate dehydrogenase (LDH), and RDW-SD affect the prognosis of patients with advanced lung cancer [4, 5]. The relationship between inflammatory indicators and postoperative stage I NSCLC is still unclear [6, 7]. The purpose of this study was to combine TNM staging with laboratory tests, general clinical data as well as postoperative pathology to predict patient prognosis.

## 2. Methods and Materials

### 2.1. Patients

**Figure 1:**
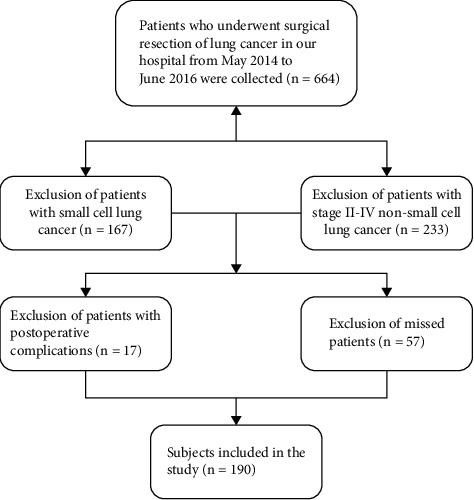
Study flow diagram of the patient population.

Patients diagnosed with stage I NSCLC who underwent radical surgery in Nanjing Chest Hospital from May 2014 to June 2016 were collected, and the follow-up deadline was June 2021. The inclusion criteria were as follows: (1) patients who have been treated with surgical resection of lung cancer; (2) patients in whom postoperative resected pathological specimens can be reevaluated; (3) patients with histological staging of postoperative resected pathological specimens based on the WHO 8th edition classification criteria; and (4) patients with pathologic stage T1-T2aN0M0 NSCLC. Patients were excluded if they met the following criteria: (1) preoperative neoadjuvant radiotherapy; (2) positive surgical margin; (3) no postoperative pathological specimens were made; (4) lymph node involvement, metastasis, or invasive chest wall; (5) history of glucocorticoid use; and (6) preoperative or postoperative infection or other bone marrow disease. A total of 190 cases were selected and the flowchart is shown in Figure 1.

This study was approved by the Ethics Committee of Nanjing Brain Hospital and was carried out in accordance with the national law and the current revised Declaration of Helsinki. Informed consent was obtained from all participants in the study.

### 2.2. Methods

Clinical data were collected as follows: gender, age, smoking history, surgical modality, tissue type, stage, degree of differentiation, chemotherapy, lymphovascular or visceral pleural invasion, PFS, OS, CEA, NLR, RDW-SD, LDH, and CYFRA211 (serum cytokeratin-19 fragment). Follow-up method: the case inquiry system and telephone follow-up were used. 190 patients were followed up every 6 months for the first 2 years after surgery and then once a year for the next 3–5 years. The last follow-up was in June 2021. The criteria for determining recurrence were as follows: the appearance of lesions at the original surgical site or the presence of new metastatic lesions (by CT, MRI, bone imaging, PET-CT, and if necessary, pathological diagnosis for clinical diagnosis). Progression-free survival (PFS) was defined as the time interval from surgery to recurrence, or death from any cause. Overall survival (OS) was defined as the number of months between the date of surgery and the date of death or last living. The normal value for CEA was <5 ug/l; the normal value for CYFRA211 was <3.3 ug/l.

### 2.3. Statistical Analysis

SPSS 25.0 software was used for statistical analysis. Categorical variables were analyzed using the chi-squared test. Survival analysis was measured by the Kaplan–Meier method. Statistically significant variables in univariate analysis were included in the Cox proportional hazards model for multivariate analysis. The optimal cutoff values of NLR, RDW-SD, and LDH were determined using a receiver operating characteristic (ROC). *P* < 0.05 was considered significant.

## 3. Results

### 3.1. Baseline Characteristics

A series of 190 consecutive patients were included in the study. The study included 92 women (48.4%) and 98 men (51.6%) with an age range of 31–79 years. Seventy (37%) patients had a history of smoking. One hundred and forty-six (76.8%) patients had adenocarcinoma, including 73 papillary carcinomas (38.4%). 140 (72.7%) patients had stage IA disease and 50 (26.3%) had stage IB. Fifty-one (26.8%) patients had poorly differentiated histology, 109 patients received adjuvant chemotherapy, visceral pleural invasion occurred in 26 patients (13.7%) and lymphovascular invasion occurred in 37 (20.5%) patients. The PFS and OS rates were 35.8% and 81.1%, respectively (Table 1).

### 3.2. Optimal Cutoff Value for ROC Curve

**Figure 2:**
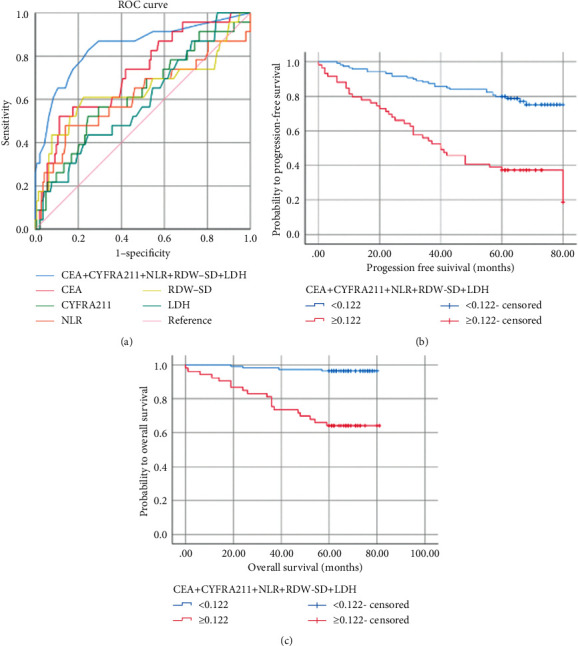
ROC curves for survival prediction. (a) Kaplan–Meier curves for progression-free survival (b) and overall survival (c) probability according to the CEA + CYFRA211 + NLR + RDW-SD + LDH level. CEA: carcinoembryonic antigen; CYFRA211: serum cytokeratin-19 fragment; NLR: neutrophil-to-lymphocyte ratio; RDW-SD: red blood cell distribution width-standard deviation; LDH: lactate dehydrogenase; AUC: area under the curve.

Based on the cutoff value of 2.840 (sensitivity: 0.500, specificity: 0.850, and AUC of the ROC curve: 0.627), patients were distributed into a high NLR group (*n* = 36) and a low NLR group (*n* = 153). A cutoff value of 43.650 fl was used to differentiate between patients with high and low preoperative RDW (RDW ≥ 43.65 fl or < 43.65 fl), with AUC of 0.655 (sensitivity: 0.625, specificity: 0.778), of which 54 (28.4%) patients was high RDW. The cutoff value for preoperative LDH levels was 191.500 u/l (sensitivity: 0.458; specificity: 0.760; AUC: 0.613). LDH ≥ 191.5 u/L was found in 55 patients (28.9%) and <191.5 u/l in 135 patients (71.1%) (Figure 2(a)). NLR, CEA, RDW-SD, LDH, and CYFRA211 were combined for diagnosis, and the cutoff value was 0.122 (sensitivity: 0.826; specificity: 0.755; AUC: 0.844), which can slightly enhance the diagnostic performance of NSCLC and was associated with the prognosis (*P* ≤ 0.001) (Figures 2(b) and 2(c)).

### 3.3. Association between Clinicopathological Characteristics and the Preoperative NLR Level

The NLR was relevant to the histological type (*P*=0.029) and gender (*P*=0.002), and an NLR ≥ 2.840 was positively correlated with men and squamous carcinoma. The rates of an elevated NLR were 13.8%, 20.0%, and 28.0% in stage IA1 + IA2, stage IA3, and stage IB, respectively, suggesting that the NLR was more likely to be elevated in patients with more advanced staging, but there was no statistically significant difference (*P*=0.232) (Table 2).

### 3.4. Prognostic Factors of PFS in Patients with Stage I NSCLC

**Figure 3:**
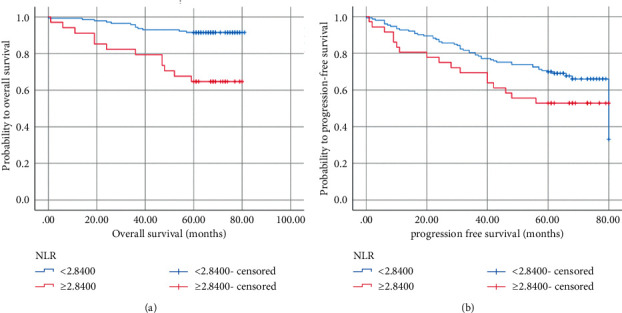
Kaplan–Meier curves for progression-free survival (a) and overall survival (b) probability according to the preoperative NLR level. NLR: neutrophil-to-lymphocyte ratio.

Univariate analysis showed that gender (*P*=0.009), smoking (*P*=0.232), histopathological type (*P*=0.002), stage (*P*=0.010), differentiation (*P*=0.002), lymphovascular invasion (*P*=0.015), CEA (*P* ≤ 0.001), CYFRA211 (*P*=0.003), and RDW-SD (*P* ≤ 0.001), and LDH (*P*=0.005) were all correlated with PFS of stage I NSCLC. There was no statistical significance between the NLR and PFS in stage I NSCLC (*P*=0.090) (Figure 3(a)). Chemotherapy had no correlation with PFS in stage I lung cancer (*P* > 0.05). Multivariate analysis suggested that lymphovascular invasion (*P*=0.027), RDW-SD (*P*=0.001), and CEA (*P* ≤ 0.001) were independent prognostic factors for PFS of stage I NSCLC (Table 3).

### 3.5. Prognostic Factors of OS in Patients with Stage I NSCLC

In univariate analyses, age (*P*=0.047), gender (*P*=0.011), smoking (*P*=0.034), pathological type (*P* ≤ 0.001), stage (*P*=0.041), differentiation (*P*=0.004), tumor location (*P*=0.006), CEA (*P* ≤ 0.001), RDW-SD (*P* ≤ 0.001), LDH (*P*=0.034), and NLR (*P* ≤ 0.001) were all associated with OS in stage I NSCLC. An elevated NLR was negatively correlated with OS (Figure 3(b)). Chemotherapy was not an independent prognostic factor for OS in stage I lung cancer (*P*=0.230). Multivariate analysis demonstrated that NLR (*P*=0.016), RDW-SD (*P*=0.004), and CEA (*P*=0.011) significantly affected OS (Table 3).

### 3.6. Establishing Risk Groups Based on Independent Prognostic Factors

According to independent prognostic factors, risk groups were divided into a low-risk group with 0 to 1 high-risk factor; an inter-moderate risk group with 2 high-risk factors; a high-risk group with 3 high-risk factors.

**Figure 4:**
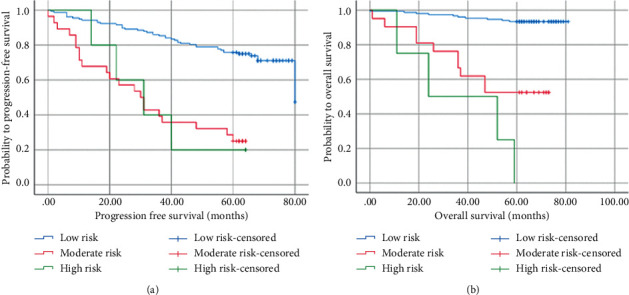
Kaplan–Meier curves for progression-free survival (a) and overall survival (b) probability according to the risk groups.

Recurrence risk groups were established as follows: 157 in the low-risk group, 28 in the intermediate-risk group, and 5 in the high-risk group. The median survival times among patients in the low-, intermediate-, and high-risk groups were 67.2, 33.2, and 34.2 months, respectively (*P* ≤ 0.001). There were significant differences between the low-risk group and intermediate-high-risk groups, while no statistical difference was found between the intermediate-risk and high-risk groups (*P*=0.967), indicating that PFS was shorter in patients with ≥2 high-risk factors (Figure 4(a)).

Death risk groups were established as follows: 152 in the low-risk group, 21 in the intermediate-risk group, and 4 in the high-risk group, with a median survival of 77.8, 51.3, and 36.5 months, respectively (*P* ≤ 0.001). There was a significant difference between the low-risk group and intermediate-high groups, but no difference between the intermediate-risk and high-risk groups (*P*=0.092), indicating that patients with ≥2 high-risk factors had shorter OS (Figure 4(b)).

## 4. Discussion

This finding suggested that lymphovascular invasion, RDW-SD, and CEA were independent prognostic factors for PFS; NLR, RDW-SD, and CEA were independent prognostic factors for OS in stage I NSCLC. An elevated NLR was associated with histological type, gender, poor OS, and not PFS in stage I NSCLC patients. The risk model showed that patients with ≥2 risk factors had a poor prognosis.

Wang et al. reported that 5-year OS was 87.6% (*n* = 265) in the observation group and 82.4% in the adjuvant chemotherapy group for stage IB NSCLC patients [8]. In a randomized controlled trial, 5-year OS was 62% and 63% in the nonchemotherapy and chemotherapy groups, respectively, with no significant difference between the two groups [9]. The guideline for postoperative resection of stage I-IIIB non-small-cell lung cancer (version 2021) stated that adjuvant therapy was not recommended after stage IA surgery but for stage IB with high-risk factors [10]. Our study showed chemotherapy was not an independent factor of patients with stage I NSCLC, which may be related to the small sample size and lack of genetic testing. Therefore, a large number of prospective studies are still needed to validate whether patients with stage I NSCLC can benefit from chemotherapy.

Our study showed that the NLR was associated with OS (*P* ≤ 0.001), which is consistent with previous studies [11]. A retrospective study demonstrated that a high NLR (≥5) was correlated with decreased RFS in early NSCLC; the more advanced the tumor stage, the higher the mean value of NLR, suggesting a higher inflammatory status and immunosuppression in advanced NSCLC; however, our study suggested that there was no significant difference between the NLR and PFS (*P*=0.086), which may be related to the small sample size [12, 13]. The increase in the ratio of tumor-associated neutrophils (TANs) and the NLR suggests a poor prognosis. The possible mechanism is that neutrophils participate in the formation of tumor cell microenvironment by secreting various cytokines and chemokines, releasing reactive oxygen species (ROS), and forming neutrophils extracellular trap (NET), which promotes tumor cell differentiation, metastasis and invasion, neovascularization, and immunosuppression, and lymphocytes promote cytokine apoptosis and inhibit tumor cell differentiation [14–16].

The previous study which included 1387 postoperative patients with stage I NSCLC indicated that lymphovascular invasion was an adverse factor for postoperative recurrence and death, and Mollberg et al. and Mitchell et al. similarly indicated that stage I lung cancer with positive lymphovascular invasion had a poor prognosis, which was in agreement with the results of our study [17–19]. RDW-SD ≥43 fl was negatively correlated with NSCLC survival [20]. Recent studies have found that stage I NSCLC with elevated RDW-SD had short PFS and OS, which was similarly confirmed in our current study [21, 22]. Elevated RDW-SD reflected a severe dysregulation of erythroid homeostasis, including abnormal erythropoiesis, which was attributed to multiple metabolic abnormalities such as oxidative stress, inflammation, malnutrition, erythrocyte fragmentation, and altered erythropoietin function [23]. CEA is related to the diagnosis and survival prediction of lung cancer, especially for adenocarcinoma and advanced patients among NSCLC [24–26]. In 378 patients with stage IA NSCLC, the multivariate analysis suggested that elevated CEA was not associated with 5-year OS, which is inconsistent with the results of the current study and may be related to the small sample size and different selected populations [27].

The limitations of this study are as follows: first, the study is retrospective and biased with a small number of patients incorporated. Second, the study did not include CA199, albumin-to-globulin ratio, and lymphocyte-to-monocyte ratio, which will be further analyzed in subsequent studies, and the optimal threshold value for the NLR remains controversial due to the different staging of selected patients and testing techniques. Last, most studies of the NLR were performed in peripheral circulating blood and whether it was consistent with neutrophils and lymphocytes in tumors remains to be further confirmed. In conclusion, the present study suggested that the NLR is an independent prognostic factor of stage I NSCLC, but it still needs to be confirmed by a large number of randomized multicenter, prospective trials.

## Figures and Tables

**Table 1:** Baseline characters of the 190 patients.

Variable	*N* (%)
*Gender*	

Male	98 (51.6)
Female	92 (48.4)

*Age at diagnosis(years)*	
≥65	69 (36.3)
<65	121 (63.7)

*Smoking history*	
Smoker	70 (37)
Nonsmoker	119 (63)

*Surgical resection*	
Sublobar resection	8 (4.2)
Lobectomy	181 (95.8)

*Histologic type*	
Adenocarcinoma	146 (76.8)
Lepidic-predominant	20 (10.5)
Acinar-predominant	30 (15.8)
Papillary-predominant	73 (38.4)
Micropapillary-predominant	3 (1.6)
Solid-predominant	12 (6.3)
Adenocarcinogenesis	8 (4.2)
Squamous	26 (13.7)
Others	18 (9.5)

*Stage*	
IA1	15 (7.9)
IA2	80 (42.1)
IA3	45 (23.7)
IB	50 (26.3)

*Differentiation*	
Poor	51 (26.8)
Moderate	38 (20.0)
Well	97 (51.1)
Unknown	4 (2.1)

*Chemotherapy*	
Yes	109 (57.4)
No	81(42.6)

*Tumor location*	
Upper lobe	118 (62.1)
Middle lobe	21 (11.1)

*Visceral pleural invasion*	
Absent	164 (86.3)
Present	26 (13.7)

*Lymphovascular invasion*	
Absent	151 (79.5)
Present	37 (20.5)

*Recurrence*	
Yes	68 (35.8)
No	122 (64.2)

*Survival*	
Yes	154 (81.1)
No	24 (12.6)
Unknown	12 (6.3)

*CEA (ng/ml)*	
≥5	37 (19.5)
<5	143 (75.3)

*CYFRA211 (ng/ml)*	
≥3.3	43 (22.6)
<3.3	138 (72.6)

*NLR*	
≥2.84	36 (18.9)
<2.84	153 (80.5)

*RDW-SD (fl)*	
≥43.65	54 (28.4)
<43.65	135 (71.1)

LDH (u/l)	
≥191.5	55 (28.9)
<191.5	135 (71.1)

**Table 2:** Relationship between clinical, pathological, and NLR in postoperative stage I NSCLC patients.

Variables	*N*	Low NLR (%)	High NLR (%)	*χ* ^2^	*P* value
Gender				9.979	**0.002**
Male	97	70 (72.2)	27 (27.8)		
Female	92	83 (90.2)	9 (9.8)		

Age at diagnosis (years)				0.625	0.429
≥65	68	53 (77.9)	15 (22.1)		
<65	121	100 (82.6)	21 (17.4)		

Smoking history				3.105	0.078
Smoker	70	52 (74.3)	18 (13.4)		
Nonsmoker	118	100 (84.7)	18 (15.3)		

Histologic type				7.064	**0.029**
Squamous	26	17 (65.4)	9 (34.6)		
Adenocarcinoma	145	123 (84.8)	22 (15.2)		
Others	12	8 (66.7)	4 (33.3)		

Stage				4.288	0.232
IA1	15	13 (86.7)	2 (13.3)		
IA2	79	68 (86.1)	11 (13.9)		
IA3	45	36 (80.0)	9 (20.0)		
IB	50	36 (72.0)	14 (28.0)		

Differentiation				2.350	0.309
Poor	51	38 (74.5)	13 (25.5)		
Moderate	38	33 (86.8)	5 (13.2)		
Well	96	79 (82.3)	17 (17.7)		

Chemotherapy				0.082	0.775
Yes	109	89 (81.7)	20 (18.3)		
No	80	64 (80.0)	16 (20.0)		

Visceral pleural invasion				0.001	0.980
Absent	163	132 (81.0)	31 (19.0)		
Present	26	21 (80.0)	5 (19.2)		

Lymphovascular invasion				1.236	0.266
Absent	150	119 (79.3)	31 (20.7)		
Present	39	34 (87.2)	5 (12.8)		

Recurrence				2.693	0.101
Yes	67	50 (74.6)	17 (25.4)		
No	122	103 (84.4)	19 (15.6)		

Survival				15.990	**0.001**
Yes	153	130 (85.0)	23 (15.0)		
No	24	12 (50.0)	12 (50.0)		

**Table 3:** Univariate and multivariate Cox regression analyses of PFS and OS.

Variables	PFS				OS			
	Univariate		Multivariate		Univariate		Multivariate	
	HR (95% CI)	*P*	HR (95% CI)	*P*	HR (95% CI)	*P*	HR (95% CI)	*P*
Age (≥65 vs.<65)	0.782 (0.483–1.268)	0.319			2.258 (1.011–5.040)	**0.047**	0.621 (0189–2.035)	0.431
Gender (male vs. female)	1.944 (1.181–3.200)	0.009	1.984 (0.927–4.246)	0.078	0.420 (0.188–0.937)	**0.011**	2.722 (0.568–13.045)	0.210
Smoking history (cur/for vs. never)	1.741 (1.078–2.813)	0.023	1.129 (0.515–2.475)	0.762	2.382 (1.067–5.318)	**0.034**	1.075 (0.267–4.331)	0.919
Histologic type (squamous vs. adenocarcinoma)	0.261 (0.113–0.607)	0.002	1.131 (0.489–2.620)	0.532	0.164 (0.069–0.391)	**0.001**	0.281 (0.059–1.337)	0.111
Stage (IB vs. IA1 + IA2)	2.067 (1.191–3.586)	0.010	1.590 (0.826–3.058)	0.349	2.463 (1.001–6.063)	**0.041**	1.928 (0.530–7.009)	0.319
Differentiation (poor vs. well)	1.513 (1.148–1.993)	0.002	0.669 (0.313–1.429)	0.578	3.918 (1.542–9.956)	**0.004**	1.122 (0.308–4.087)	0.862
Lymphovascular invasion (present vs. absent)	1.948 (1.138–3.333)	0.015	2.100 (1.090–4.043)	**0.027**	1.105 (0.413–2.961)	0.842		
Visceral pleural invasion (absent vs. present)	0.969 (0.480–1.957)	0.930			0.556 (0.131–2.364)	0.427		
Tumor location (upper vs. lower)	1.476 (0.851–2.562)	0.166			3.220 (1.391–7.453)	**0.006**	2.114 (0.765–5.843)	0.149
Chemotherapy (no vs. yes)	1.754 (0.955–3.222)	0.070			1.681 (0.720–3.929)	0.230		
CEA (high vs. low)	3.895 (2.358–6.435)	0.001	2.953 (1.637–5.329)	**0.001**	5.004 (2.206–11.351)	**0.001**	3.995 (1.377–11.593)	**0.011**
CYFRA211 (high vs. low)	2.160 (1.306–3.571)	0.003	1.795 (0.879–3.664)	0.108	2.192 (0.929–5.172)	0.073		
RDW-SD (high vs. low)	3.003 (1.863–4.841)	0.001	2.570 (1.458–4.528)	**0.001**	5.110 (2.234–11.686)	**0.001**	4.638 (1.6614–13.326)	**0.004**
LDH (high vs. low)	2.009 (1.236–3.26	0.005	1.622 (0.904–2.912)	0.105	2.385 (1.069–5.325)	**0.034**	1.810 (0.634–5.168)	0.268
NLR (high vs. low)	1.612 (0.929–2.800)	0.090			4.839 (2.172–10.780)	**0.001**	3.208 (1.243–8.277)	**0.016**

## Data Availability

All data generated or analyzed during this study are included in this published article. The data could be freely available for anyone interested.
